# Pulmonary Vein Isolation in Obese Compared to Non-Obese Patients: Real-Life Experience from a Large Tertiary Center

**DOI:** 10.3390/jcdd9080275

**Published:** 2022-08-17

**Authors:** Julian Wolfes, Daniel Hoppe, Christian Ellermann, Kevin Willy, Benjamin Rath, Patrick Leitz, Fatih Güner, Julia Köbe, Philipp S. Lange, Lars Eckardt, Gerrit Frommeyer

**Affiliations:** Department of Cardiology II (Electrophysiology), University Hospital Münster, Albert-Schweitzer-Campus 1, 48149 Münster, Germany

**Keywords:** atrial fibrillation, obesity, pulmonary vein isolation, risk factors

## Abstract

1. Introduction: Pulmonary vein isolation (PVI) is an established procedure used to achieve rhythm control in atrial fibrillation (AF). In obese patients (pts), in whom AF occurs more frequently, a reduced effectiveness of PVI has been observed. Therefore, this study’s aim was to compare the long-term efficacy of PVI between obese and non-obese patients. 2. Methods: We enrolled 111 consecutive pts with a body mass index (BMI) of >30 kg/m^2^ undergoing PVI from our large registry. Procedural data and outcomes were compared with a matched group of 115 non-obese PVI pts and the long-term outcomes were analyzed. 3. Results: Overall follow-up duration was 314 patient-years in the obese and 378 patient-years in the non-obese group. The follow-up rate was 71% in the obese and 76% in the non-obese group. In both groups, their AF-characteristics did not differ significantly, while known risk factors were significantly more prevalent in the obese group. Procedural characteristics were similar in both groups. During follow-up, the obese pts demonstrated significant weight loss compared to the non-obese pts, while at the same time, the overall recurrence rate during follow-up did not differ significantly between both groups (obese: 39.2% and non-obese: 43.7%). PVI related and long-term complications were comparable between both groups. In the univariate analysis, obesity was not found to be associated with an increased AF recurrence risk. 4. Conclusion: These real-life data demonstrate that obese pts may not show higher AF recurrence rates after PVI compared to pts with normal body weight. Furthermore, PVI was found to be safe and effective in obese patients; thus, a BMI alone may not be a criterion for refusal of PVI.

## 1. Introduction

Obesity represents a key challenge for health systems worldwide [1]. The development of obesity has reached epidemic proportions. The association between obesity and various cardiovascular diseases such as coronary artery disease, high blood pressure, and diabetes mellitus, as well as atrial fibrillation (AF) has been proven [2]. Obese patients show AF more frequently, are associated with a further limitation of exercise capacity, and tend to show persistent forms of AF more often than non-obese patients [3,4].

While rhythm control of AF primarily serves to control symptoms, new studies question this by pointing to a prognostic benefit of a rhythm control strategy [5]. Despite the above-mentioned coherence of studies on the subject of obesity and cardiovascular disease, there are contradicting data on complication rates and the short- and long-term effectiveness of pulmonary vein isolation with regard to rhythm maintenance in obese patients. While some authors report reduced chances of success [6,7,8,9] and/or increased complication rates, other authors report an equivalent effectiveness of PVI without increased complication rates [10,11,12]. It is noteworthy that the difference in outcomes is partly based on the comparison of different ablation procedures or patient groups and morbidity burden variation [9].

The following study aims to examine the effectiveness of pulmonary vein isolation with different techniques in a matched group analysis of a real-life collective, in which both different types of atrial fibrillation (paroxysmal and persistent) and different ablation methods are represented.

## 2. Methods

The study was approved by the local ethics committee and complies with the Declaration of Helsinki. A retrospective analysis of our prospectively conducted PVI registry between May 2011 and May 2020 was performed. In total, 111 consecutive patients with a BMI of >30 kg/m^2^ at the time of the procedure were identified. Patient characteristics (e.g., history, AF-pattern, AF-duration, comorbidities, antiarrhythmic medication, etc.), procedural characteristics (e.g., number of previous ablation procedures, mode of PVI, procedure times, radiation dose, etc.), periprocedural complications, acute success, and duration of hospital stay were analyzed. We used a propensity matched design for procedural characteristics, AF-type, and history with a group of 115 PVI patients from our registry with a BMI of <30 kg/m^2^ and compared intraprocedural and postprocedural outcomes. All obese pts were taught about the significance of obesity for AF and recommended to reduce weight after the ablation procedure. The patients were encouraged to perform regular ECG check-ups (e.g., 3-, 6-, and 12-months following ablation) at the general practitioner. In a case of a suspected AF recurrence, the ECG was reviewed by an electrophysiologist. All patients were contacted for a follow-up telephone interview between August 2020 and May 2021 to define meantime AF-related complications, quality of life, actual body weight, and AF relapses defined as a documented AF > 30 s using a 12-lead ECG.

For the statistical analysis, SPSS software (version 27.0; SPSS Inc., Chicago, IL, USA) was employed. The student t-test was employed for metric variables. The chi-square test and exact Fisher test were applied for the comparison of proportions between the groups. To determine independent predictors of AF recurrence during follow-up, binary logistic regression models were used, while AF recurrence (yes; no) was set as the dependent variable. A *p* value < 0.05 was considered statistically significant.

## 3. Results

### 3.1. Patient Characteristics

**Figure 1:**
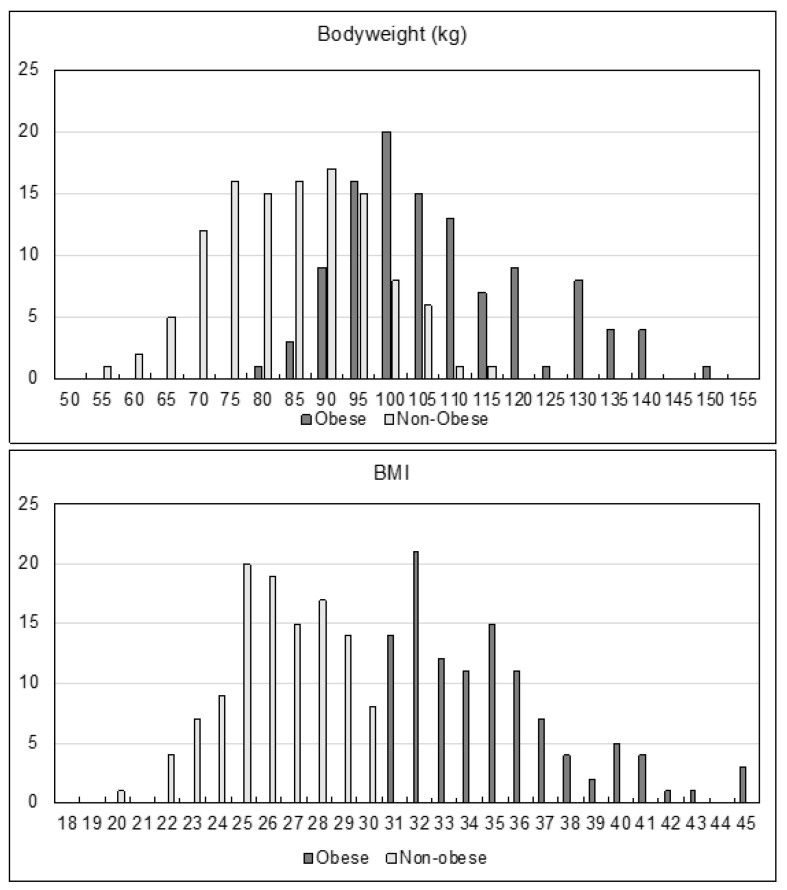
Histogram of patient’s body weight and BMI at baseline.

The AF characteristics and the procedural characteristics did not differ significantly between both groups (Table 1 and Figure 1). Persistent AF was present in nearly half of both groups. Furthermore, the use of single-shot devices did not differ significantly between both groups (Cryoballoon (Medtronic Arctic Front): 50.5% in the obese group vs. 58.3% in the non-obese (*p* = 0.23); Multielectrode Pulmonary Vein Ablation Catheter (Medtronic PVAC Gold): 27% in the obese group vs. 26.1% in the non-obese (*p* = 0.881)). Radiofrequency ablation was performed with the guidance of a 3D-mapping system (Abbott EnSite NavX). Repeat ablation procedures represented nearly a quarter in both groups (21.6% in the obese group vs. 20.9% in the non-obese (*p* = 0.87)). Regarding patient characteristics, age and gender did not differ significantly between the groups. Furthermore, left ventricular ejection fractions (LVEFs) and thyroid disorders were equal in both groups. On the other hand, comorbidities associated with obesity such as hypertension, coronary artery disease, diabetes mellitus, and sleep apnea were found significantly more often in the obese group.

Furthermore, obese patients had antiarrhythmic medication significantly more often prior to PVI and a previous electrical cardioversion.

### 3.2. Intrahospital Outcome

Neither the procedural duration nor the fluoroscopic time differed significantly (Table 2). The dose area product was more than twice as high in the group of obese patients. When focusing on the procedural characteristics within the groups, RF-ablations were more time-consuming than the use of “single-shot” devices (PVAC or cryoballoon), while the cryoballoon procedure especially was associated with significantly less radiation in both groups (Table 3). Complete isolation at the end of the procedure succeeded equivalently in both groups. Complications were very rare in obese and non-obese patients and none of them showed any statistically significant differences in distribution. No patient died during the hospital stay. Pericardial effusion tended to be more common in the non-obese group. Pericardial tamponade occurred once in both groups, and pericardial drainage was uncomplicated for both patients. Femoral bleeding, all of which could be managed conservatively, tended to occur more frequently in the obese group. The incidence of non-sustained phrenic nerve palsy did not differ significantly. Pulmonary vein stenosis or thromboembolic complications did not occur in any patient post-procedurally. The number of intrahospital AF recurrences was also not significantly different with 17.1% in the obese group and 14.8% in the non-obese group (*p* = 0.717). The rates of postprocedural antiarrhythmic drug prescriptions differed significantly between both groups as 79.3% of the overweight patients received an AAD, while only 63.5% of the non-obese patients received an AAD (*p* < 0.05). Considering the significantly raised pre-procedural AAD rate in the obese patients, around a quarter of the patients in both groups (25.2% of obese and 23.5% of non-obese) received a new antiarrhythmic medication. The duration of the in-hospital stays differed from 3.05 ± 1.77 nights for the obese patients and 2.77 ± 1.39 nights for the non-obese, without reaching the threshold for statistical significance.

### 3.3. Long-Term Follow-Up

Overall, the follow-up duration was 314 patient-years in the obese and 378 patient-years in the non-obese group. The average follow-up duration per patient was 3.98 ± 1.97 years in the obese and 4.34 ± 1.96 years in the non-obese group.

The AF recurrence rate did not differ significantly between the two groups (39.2%: obese vs. 43.7%: non-obese (*p* = 0.637)). There was also no significant difference in stroke rate with one stroke per group (Table 4). Satisfaction with the procedure tended to be higher in the non-overweight group, without reaching the threshold for statistical significance. In the follow-up period, a slightly larger proportion of non-obese patients received a repeat PVI, whereas cardioversion tended to be performed more frequently in the obese patient group. During follow-up, the statistically significant differences in body weight remained stable. However, the obese patients achieved significant weight loss compared to the non-obese patients.

### 3.4. Risk Factors for AF Recurrence

In the univariate analysis, obesity was not found to be a risk factor for AF recurrence (Table 5). In the whole collective, the risk factors for AF recurrence were found to be thyroid disorder, smoking, and intrahospital AF recurrence, while cryoablation was found to be associated with reduced AF recurrence during follow-up. When focusing on obese patients, thyroid disorders were solely associated with a significantly increased AF recurrence rate. In the non-obese collective, smoking was found to be associated with AF recurrence.

## 4. Discussion

The results of the present real-life study suggest the following conclusions: (1) AF recurrence rates after PVI are not higher in obese patients than in non-obese. (2) AF ablation is effective and safe for obese patients. (3) Obese patients tend to show a slight body weight reduction after PVI compared to non-obese patients. (4) Obese PVI patients do have more cardiovascular risk factors than non-obese patients. (5) AAD prescriptions are more frequent in obese patients pre- and post-PVI, but both collectives show a similar initial increase in AAD prescriptions post-PVI. (6) The radiation dose is significantly higher in obese patients.

### 4.1. AF Recurrence Rates after PVI

Previous studies compared PVI in obese with non-obese patients with heterogeneous outcomes concerning AF recurrence. Two studies evaluating cryoablation in obese patients did not show significantly raised recurrence rates in obese patients [11,12], whereas studies with mixed ablation techniques [8] and a study employing radiofrequency ablation [7] showed higher recurrence rates in obese patients. Furthermore, a study by Winkle et al. which employed a mixed collective of primary and repeat ablation demonstrated reduced ablation success in obese patients. In this study, significantly more patients with persistent AF were obese [9]. However, the present study is the first to show equal AF recurrence rates in a mixed collective of different ablation techniques, different AF patterns, and also including repeat ablation procedures with a very long follow-up. This effect might partly be based on a very long inclusion period. In the univariate analysis, no ablation technique was significantly superior in maintaining sinus rhythm, although the study might be underpowered to evaluate this.

### 4.2. Ablation Safety

We did not observe increased complication rates in the obese patients following AF ablation. Many examiners fear pericardial effusions, especially in overweight people, due to the expected more difficult access route for pericardiocentesis. However, this concern is not supported by published data; in addition, our collective showed a higher rate of pericardial effusions in the non-obese patients, which is an observation that may potentially be explained by a thinner myocardial wall thickness.

### 4.3. Body Weight Development Following PVI

A significant body weight reduction during follow-up was observed in the obese group in the present study. Whether an increased physical fitness in sinus rhythm may have contributed to body weight reduction remains speculative. Furthermore, data do not allow an evaluation of lifestyle interventions after the hospital stay. However, encouragements to increase fitness and reduce body weight from the medical team during the hospital stay may also have significantly contributed to the weight loss.

### 4.4. Risk Factors in Obese Patients

Obesity is associated with cardiovascular risk factors in our collective as in the general population, and most of these risk factors are generally associated with reduced ablation success [13]. However, no risk factor alone besides thyroid disorders led to a significantly raised recurrence rate in the univariate analysis.

### 4.5. Antiarrhythmic Drugs in Obese Patients

Obese patients received antiarrhythmic drugs (AAD) significantly more often than non-obese patients, but the rate of newly prescribed AAD post-PVI was equal in both groups. As a result, the observed similar AF recurrence rate in both groups might not only be attributable to PVI, but also to pharmaceutical interventions. Nonetheless, the combination of medical and interventional AF treatment represents a synergistic state-of-the-art treatment and is supported by the recently published EAST study in which the majority of patients received AADs [5].

### 4.6. Radiation and Operator Safety

Our study showed significantly raised doses of radiation with equal procedure times in the obese patient group. This has been formerly reported and is due to automatically adjusted tube voltage and tube current based on the patients’ attenuation [14]. Previous investigations showed that obese patients tend to cause more radiation scatter [15]. Therefore, radiation protection for the patient and the investigator is extremely important with obese patients.

## 5. Clinical Implications and Limitations

This study shows that in a real-world collective with mixed AF patterns and different AF ablation techniques, the decision for or against AF ablation should not be based on BMI alone as obese patients do not show more in-hospital complications or higher AF recurrence rates during follow-up. Furthermore, obese patients might profit from PVI in terms of restoring sinus rhythm. Based on the univariate analysis, obese patients with thyroid disorder should be evaluated more critically for AF ablation and optimal endocrine treatment should be pursued. The limitations of the above-mentioned data are based on the retrospective nature of the study and the fact that the patients were contacted by telephone only. As we did not investigate COVID infections during follow-up, a potential interaction with the COVID pandemic cannot be excluded. The lost to follow-up rate was significant; nonetheless, the equal rate in both groups points against a body weight-dependent bias. Furthermore, another well-recognized study showed an equal lost to follow-up rate [16].

## Figures and Tables

**Table 1:** Patient characteristics at baseline.

Baseline Characteristics			
	Obese (*n =* 111)	Non-Obese (*n =* 115)	*p*-Value
Age	61.0 ± 9	59.3 ± 12.9	0.071
Female sex	47 (42.3%)	38 (33%)	0.17
Bodyweight (kg)	106.7 ± 14.7	82.8 ± 11.9	0.01
BMI (kg/m^2^)	34.34 ± 3.4	25.9 ± 2.2	0.01
Structural heart disease	43 (38.7%)	26 (22.6%)	0.01
Hypertension	94 (84.7%)	58 (50.4%)	0.01
Diabetes mellitus	24 (21.6%)	8 (7%)	0.002
OSAS	17 (15.3%)	4 (3.5%)	0.002
Coronary artery disease	25 (22.5%)	14 (12.2%)	0.052
LVEF	57.5 ± 6.8	59 ± 5.3	0.085
Smoking	35 (31.5%)	20 (17.4%)	0.02
Thyroid disorder	22 (19.8%)	25 (21.7%)	0.746
Persistent AF	61 (55%)	57 (49.6%)	0.428
Antiarrhythmic drugs before PVI	60 (54.1%)	46 (40%)	0.045
Previous cardioversion	65 (58.6%)	48 (41.7%)	0.016
LA-dilatation	64 (57.5%)	36 (31.3%)	0.000093
Repeat ablation	24 (21.6%)	24 (20.9%)	0.872

**Table 2:** Procedural and intrahospital characteristics.

Procedural and Intrahospital Characteristics			
	Obese (*n =* 111)	Non-Obese (*n =* 115)	*p*-Value
RF-ablation	25 (22.5%)	18 (15.7%)	0.477
Cryoballoon	56 (50.5%)	67 (58.3%)	0.23
PVAC	30 (27%)	30 (26.1%)	0.881
Repeat ablation	24 (21.6%)	24 (20.9%)	0.872
EP duration (min)	117.4 ± 47.7	108.5 ± 43.9	0.146
Impulses	10.3 ± 11.2	10.5 ± 8.2	0.891
Flouroscopy time (min)	18.3 ± 7.9	16.1 ± 7.8	0.04
DAP-Value (cGy/cm^2^)	2360.83 ± 1759.6	989.75 ± 756.2	0
Complete isolation	106 (95.5%)	112 (97.4%)	0.493
Death	0	0	1
Pericardial effusion	3 (2.7%)	6 (5.2%)	0.5
Pericardial tamponade	1 (0.9%)	1 (0.9%)	1
Embolism	0	0	1
Phrenic palsy	2 (1.8%)	1 (0.9%)	0.617
Femoral bleeding	5 (4.5%)	2 (1.7%)	0.276
Intrahospital AF recurrence	19 (17.1%)	17 (14.8%)	0.717
Antiarrhythmic drugs after PVI	88 (79.3%)	73 (63.5%)	0.012
Hospital stay (nights)	3.05 ± 1.77	2.77 ± 1.39	0.199

**Table 3:** Intraprocedural details regarding ablation technique and resulting EP characteristics.

Intraprocedural Details				
Obese				
	n	EP Duration (min)	Flouroscopy Time (min)	DAP-Value (cGy/cm^2^)
RF-ablation	25	175.2 ± 52.71	24.16 ± 21.95	3327.67 ± 1800.14
PVAC	30	109.47 ± 25.3	16.04 ± 5.49	2148.4 ± 1595.73
Cryoballoon	56	95.81 ± 30.72	15.85 ± 5.79	2026.94 ± 1708.85
Non-obese				
	n	EP Duration (min)	Flouroscopy Time (min)	DAP-Value (cGy/cm^2^)
RF-ablation	18	169.44 ± 46.49	22.21 ± 9.79	907.75 ± 673.83
PVAC	30	118.63 ± 40.81	17.67 ± 9.71	1261.73 ± 935.13
Cryoballoon	67	87.57 ± 23.3	13.78 ± 4.83	901.14 ± 677.71

**Table 4:** Long-term follow-up of obese and non-obese patients following PVI.

Long Term Follow-Up			
	Obese (*n =* 79)	Non-Obese (*n =* 87)	*p*-Value
AF recurrence	31 (39.2%)	38 (43.7%)	0.637
Stroke	1 (1.3%)	1 (1.1%)	1
Confidence with ablation	69 (87.3%)	82 (94.2%)	0.175
Electrical cardioversion during follow-up	6 (7.6%)	3 (3.4%)	0.312
Repeat ablation during follow-up	18 (22.8%)	23 (26.4%)	0.595
Actual bodyweight	100 ± 16,2	83.9 ± 11.9	<0.001
Bodyweight compared to procedure date	–4.8 ± 8.8	0.9 ± 6.3	<0.001

**Table 5:** Univariate analysis for risk factors of AF recurrence following PVI in obese and non-obese patients.

Riskfactors of AF Recurrence						
	All		Obese		Non-Obese	
Variable	Coefficient	*p*-Value	Coefficient	*p*-Value	Coefficient	*p*-Value
Obesity	–0.117	0.474				
Bodyweight	0.002	0.726	−0.002	0.749	0.004	0.755
BMI	–0.003	0.871	0.017	0.535	–0.029	0.508
Female sex	0.152	0.220	0.015	0.930	0.192	0.480
Age	0.005	0.355	0.007	0.551	0.005	0.527
Persistent AF	0.100	0.347	–0.030	0.863	0.291	0.162
AF duration	–0.005	0.574	–0.015	0.439	–0.008	0.571
Previous cardioversion	0.030	0.776	0.026	0.874	0.201	0.289
Structural heart disease	–0.097	0.397	0.021	0.892	0.014	0.951
Hypertension	0.224	0.063	0.361	0.103	0.089	0.634
Diabetes mellitus	–0.195	0.270	–0.271	0.208	–0.041	0.923
OSAS	0.044	0.768	0.115	0.624	–0.358	0.366
Coronary artery disease	0.075	0.597	0.195	0.278	–0.303	0.346
Thyroid disorder	0.342	0.003	0.542	0.006	0.032	0.882
Smoking	0.231	0.044	0.092	0.571	0.431	0.047
Repeat ablation	–0.055	0.663	–0.205	0.291	0.071	0.754
Cryoballoon	–0.310	0.015	–0.356	0.215	–0.329	0.138
RF ablation	0.177	0.323	0.270	0.299	0.110	0.748
PVAC	0.191	0.130	0.135	0.533	0.220	0.335
AAD before PVI	0.147	0.167	0.242	0.142	–0.083	0.641
AAD after PVI	0.073	0.518	0.325	0.142	–0.011	0.948
Intrahospital AF recurrence	0.287	0.013	0.340	0.062	0.231	0.255

## Data Availability

The data underlying this article will be shared on reasonable request to the corresponding author.
